# Early Auditory Processing Predicts Efficient Working Memory Functioning in Schizophrenia

**DOI:** 10.3390/brainsci12020212

**Published:** 2022-02-03

**Authors:** Oded Meiron, Jonathan David, Asaf Yaniv

**Affiliations:** 1 Clinical Research Center for Brain Sciences, Herzog Medical Center, Jerusalem 91035, Israel; yonidavid9@gmail.com (J.D.); asafyaniv@gmail.com (A.Y.); 2 Faculty of Education, Bar-Ilan University, Ramat Gan 5290002, Israel

**Keywords:** dorsolateral prefrontal cortex (DLPFC), executive attention (EA), mismatch negativity (MMN), negative symptoms

## Abstract

Early auditory processing (EAP) deficits have been consistently documented in individuals diagnosed with schizophrenia (SZ). However, a relationship between EAP and executive attention has not been confirmed in SZ versus healthy controls (HC). The current study aimed to demonstrate that unlike HC, in SZ patients, auditory change-detection event-related potentials (ERPs) are significantly associated with executive working memory (WM) functioning. Additionally, correlational analyses investigated the relationships between patients’ auditory ERPs, WM performance, and schizophrenia symptom severity scores. We examined verbal WM accuracy associated with “executive-control” prefrontal cortex mechanisms and EAP ERPs under midline prefrontal electrodes in 12 SZ patients versus 12 demographically matched HC. Mismatch negativity (MMN) amplitudes and latencies in SZ patients were not significantly different from HC, however, their verbal WM performance was significantly impaired versus HC. Importantly, prolonged MMN latencies in the SZ group were correlated with better WM accuracy. In the HC group, WM accuracy was unrelated to MMN latencies. Patients’ MMN parameters were unrelated to schizophrenia symptom-domain severity. However, patients’ WM RTs and accuracy were significantly related to illness severity and negative symptom severity, respectively. Therefore, inefficient sensory excitation related to EAP timing may underlie poor executive verbal WM functioning and might indirectly exacerbate the severity of negative symptoms in SZ. Treatments targeting prefrontal cortex dysfunction in schizophrenia are discussed.

## 1. Introduction

Schizophrenia (SZ) is a neurodevelopmental disorder involving neurophysiological abnormalities, a complex and heterogeneous set of positive and negative symptoms, cognitive impairments, and pervasive dysexecutive behaviors directly associated with dorsolateral prefrontal cortex (DLPFC) hypofunction [1,2,3]. Negative symptoms, which include dysexecutive behaviors (e.g., emotional withdrawal, difficulty in abstract thinking) may result from executive attention (EA) deficits that disrupt the ability for coherent “reality monitoring” linked to DLPFC dysfunction and poor verbal working memory (WM) performance [2,4,5].

It has been suggested that EA impairments and negative symptoms in SZ both represent a failure in DLPFC engagement during executive WM operations [2,3], and, alternatively, may be affected by abnormal bottom-up sensory neural activity (e.g., reduced evoked MMN amplitudes) mediated via changes in temporal-frontal cortex N-methyl-D-aspartate (NMDA) receptor excitability [6,7]. However, in comparison to SZ patients’ impaired EA functioning (e.g., poor WM performance), HC EA functioning is possibly independent of early sensory cortex excitability, since their EA responses (i.e., working memory accuracy) are driven mainly by top-down prefrontal networks that efficiently integrate encoded multidimensional information (DLPFC heteromodal association cortex processing data from multiple sensory modalities [8]) prior to initiating a goal-directed response [2,9]. Unlike SZ patients, HC heteromodal prefrontal cortex mechanisms are highly active in conditions requiring increased EA involvement, promoting enhanced prefrontal cortex functional inhibition (mediated by power changes in alpha oscillations [10]) of excessive activity in modality-specific WM networks and allowing for accurate WM responses [2]. In contrast to HC, SZ patients’ modality-specific prefrontal cortex mechanisms are impaired [2,11], possibly stemming from diminished early auditory information processing (EAP) in SZ [7,12]. However, although MMN excitability is generally diminished in SZ versus HC, associations between MMN generation and DLPFC-mediated cognitive control in SZ have not been investigated robustly, particularly in cognitively taxing conditions requiring enhanced EA control of encoded verbal WM multimodal competing targets.

Event-related electroencephalography (EEG) assessments of pre-attentive mismatch negativity (MMN) auditory event-related potentials (ERPs) generated at the primary auditory cortex (and noted at midline prefrontal electrodes), which denote transient echoic memory changes, have been reported to correlate with glutamatergic NMDA receptor functioning [6,7,13,14]. Impaired echoic memory change-detection mechanisms related to abnormal NMDA receptor activity may also reflect a transient DLPFC failure to modulate pre-attentive auditory network activity in schizophrenia patients versus healthy controls (HC). Inevitably, this impairment in early “transient storage” is followed by impaired “executive functioning” responses [15]. In correspondence, verbal WM scores (dominated by DLPFC top-down executive control processes) and evoked MMN amplitudes (dominated by bottom-up sensory processes) are both reliable functional measures of DLPFC-mediated attentional-control in humans and, particularly, in individuals diagnosed with SZ [2,6,9,16]. However, the relationship between these two different prefrontal WM sub-processes (i.e., executive attention and pre-attentive auditory processing) within SZ patients versus healthy controls (HC) has yet to be ascertained. 

Multiple studies in SZ patients consistently report correlations between MMN amplitudes and cognitive functioning, indicating that attenuated MMN amplitudes predict lower executive functioning scores [13,14,17], increased negative symptom severity, and poor functional outcomes [12,13]. Still, the underlying association between pre-attentive auditory cortical network activation and executive WM dysfunction in SZ remains unclear. Thus, the current investigation evaluated whether increased top-down processing network activity within heteromodal DLPFC circuitry in SZ is abnormally sensitive to earlier sensory change-detection mechanisms [8]. Moreover, it is hypothesized that hypoactive domain-general information processing within heteromodal DLPFC mechanisms is modulated by abnormal early sensory excitability [2,7]. However, in highly demanding executive WM tasks [2,5,9], SZ patients may be forced to rely on insufficient prefrontal inhibition to suppress irrelevant early sensory excitability when attempting to activate relevant auditory cortex WM storage networks [9,18]. Hence, under certain cognitively challenging conditions, slower MMN amplitude latencies, not smaller MNN amplitudes, may underlie slower and less accurate verbal WM responses in SZ versus healthy controls.

To clarify the relationship between top-down EA responses and bottom-up pre-attentive auditory excitability in SZ versus HC, we proposed utilizing a modified EA task [5] that requires a pronounced involvement of DLPFC-mediated domain-general WM storage (i.e., spatial-verbal memory storage) in interference-rich conditions that impose multiple sources of interferences during verbal memory retrieval [2,19]. The EA task requires parallel top-down prefrontal functional inhibition of irrelevant, recently encoded verbal WM items. Thus, the current investigation utilized an “interference-rich” executive WM paradigm [2,5,9] to putatively support the idea that, during executive WM conditions requiring increased prefrontal cortex functional inhibition of early modality-specific auditory information [10], slower MMN peak-amplitude latencies will be related to efficient verbal WM responses (i.e., higher accuracy), particularly in schizophrenia patients. 

In regard to disease-specific psychopathology of DLPFC-mediated top-down control of verbal WM responses in SZ, auditory MMN peak-amplitudes and latencies were hypothesized to be unrelated to schizophrenia symptom domain severity, while impaired top-down verbal WM functioning (e.g., WM accuracy) was hypothesized to be significantly related to the severity of negative symptoms. 

Therefore, the objective of the current study was to demonstrate that, unlike in HC, in SZ patients, auditory change detection event-related potentials (ERPs) are significantly associated with executive working memory (WM) functioning. Additionally, we aimed to investigate the relationships between patients’ auditory ERPs, WM performance and schizophrenia symptom severity scores.

## 2. Materials and Methods

We examined 12 SZ patients versus 12 demographically matched HC (10 males, mean age in SZ = 42.0, SD = 14.26, mean age in HC = 38.5, SD = 16.89). Informed consent was obtained from all study participants. The Medical Center’s Internal Review Board and the State Ministry of Health approved all procedures in accordance with the Declaration of Helsinki. Inclusion criteria for SZ patients included chronically medicated patients between ages 18–75 (M = 42, SD = 14.26), primary diagnosis under DSM-IV of schizophrenia or schizoaffective disorder, right-handedness, and stable doses of antipsychotic medication for ≥ 4 weeks (see Supplementary Table S1 to view description of ongoing medications and other clinical characteristics of SZ patients). Prior to experimental procedures, all SZ patients were clinically assessed for global cognitive impairment using the Mini-Mental State Examination (MMSE) and evaluated for psychosis severity using the Positive and Negative Syndrome Scale (PANSS). Patients’ mean PANSS score was 69.75 (SD = 14.6), and their mean MMSE score was 27.58 (SD = 1.78). HC were included only if they were right-handed, in healthy physical condition, and without a psychiatric or neurological history.

### 2.1. Executive Attention (EA) Task

**Figure 1:**
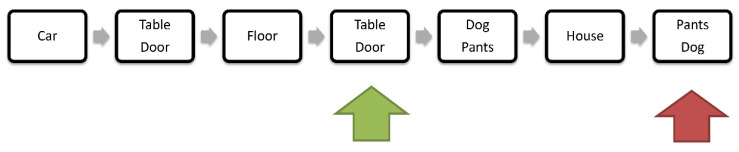
Executive attention (EA) task. Illustration depicts seven trials (black squares visually displaying single words or word pairs) during the modified computerized verbal *n*-Back task. Participants are instructed to study displayed word stimuli in order to recognize an exact stimulus that appeared two trials ago by key press response. As can be viewed in the figure, the 4th trial (marked by green arrow) in the sequence requires a key-press “GO” response for correctly recognizing the stimulus that appeared two trials ago. The 7th trial (marked by red arrow) requires a “NO-GO” response (like the other trials unmarked by arrows); thus, the participant is required to correctly reject the item as a target and to avoid responding to the stimulus during “NO-GO” trials. Each trial appears for one second (squares), as do the inter-trial intervals (gray arrows, indicating inter-stimulus intervals that display a white screen for 1 s). There were three rounds of 42 “GO” and 42 “NO-GO” randomly mixed trials, with 30 s resting periods between the WM task rounds.

A visual representation of a modified EA computerized task (noting verbal WM accuracy) used in the current study can be viewed in Figure 1. Briefly, the revised *n*-Back task we applied in the current study was similar to the classic *n*-Back version [5,20]. Correct key press responses (number of hits) and their reaction times (RTs for hits) were stored on the computer for prospective EA performance analysis (e.g., number of hits and their mean RTs). It was hypothesized that increased task difficulty of the current modified EA task would more effectively engage DLPFC-EA mechanisms [5]. In the current study, higher EA task difficulty was achieved mainly by enforcing a short stimuli duration (1000 ms) and by increasing the randomness of visual stimuli presentation (versus quasi-randomization in earlier EA task versions). Unlike the EA tasks used previously [19,21], in the current modified *2*-back WM task, there were considerably more trials (forcing a total 126 correct responses versus a total of 32 correct responses in the older task versions [2,19]), including shorter inter-stimulus intervals (ISI) and shorter response intervals (1000 ms). 

### 2.2. EEG Data Collection and Analysis

EEG data were collected using a PC-based Neuroscan SCAN digital data acquisition system (ASA ANT system, Hengelo, The Netherlands). Acquired electrical activity was digitized continuously via ANT amplifier (ANT, Hengelo, The Netherlands) at a sampling rate of 512 Hz with a band-pass of 0.01 to 256 Hz. EEG recordings utilized a 32 channel shielded cap (WaveGuardTM, ANT-neuro, Berlin, Germany) referenced to a common average (50 Hz notch-filter and AFz serving as ground), and placed according to the International 10–20 EEG system with impedance kept under 5 kΩ. 

The computerized MMN task consisted of approximately 495 tones. There were 347–350 standard tones (100 ms, 1000 Hz, 70%) with 0.8 s inter-stimulus intervals (ISI) and 147–153 deviant tones with 6.1 s ISIs. All tones had nominal intensity of 70 dB and 5 ms rise and fall time. Two to three extra standards were inserted randomly. The deviant tones were embedded between the standard tones and were different either in length (49–51 tones, 150 ms, 10%), volume (49–51 tones, 60 dB intensity, 10%), or pitch (49–51 tones, 1100 Hz, 10%). The MMN task was approximately 5 min long. Digitized waveforms, along with 0.1 ms timing pulses and digital stimulus identification tags, were stored on a hard drive for subsequent analysis. SZ patients and HC were asked to watch a silent movie (nature video) while trying to ignore the tones heard simultaneously through bilateral intra-aural earphones. 

EEG data were analyzed with Matlab (Mathworks, Natick, MA, USA) using the toolbox Fieldtrip (ftp://ftp.fieldtriptoolbox.org/pub/fieldtrip/, accessed on 2 January 2022) and custom-written Matlab scripts [22]. All EEG preprocessed data (including trial selection and coarse artifact detection) were re-referenced to mastoid electrodes and a band-pass filter from 0.1 to 30 Hz. The continuous data were epoched from a −0.3 to 0.5 s (800 ms ERP epochs) time-window relative to the onset of the auditory stimuli. In regard to artifact rejection, noisy channels and trials were identified and excluded by visual inspection (removing ocular and myogenic artifacts) and statistical criteria (kurtosis of amplitude values across trials and channels). Trials and channels in which amplitude exceeded ±100 µV were excluded from further analysis at the visual artifact rejection stage; this was followed by interpolation using clean data from neighboring non-rejected channels within a 4 cm radius. Across all remaining clean epochs, time-locked changes in evoked amplitude of standard pitch vs. deviant pitch were computed relative to a 250 ms pre-stimulus window and averaged, resulting in a stimulus-specific averaged ERP waveform per subject and per stimulus condition (e.g., standard versus odd sound). Following averaging of all clean epochs per subject of standard versus deviant ERP waveforms, averaged MMN amplitudes per subject were obtained by point-by-point digital subtraction of the averaged standard stimulus waveform values from average waveform values elicited by the deviant stimuli. Afterwards, the peak negativity values of each difference waveform within a 50–200 ms post-stimulus window [23] (relative to 100 ms pre-stimulus window) were extracted (i.e., averaged from MMN peak amplitude and peak-amplitude latencies) and saved for later statistical analysis. In both groups, ERP waveforms were inspected to verify that MMN was consistently present under midline Fz, Cz and Pz electrodes. We used SPSS 25 software (IBM, Armonk, NY, USA) for all statistical analyses. Derived peak MMN amplitudes and their latencies for deviant pitches at frontal-midline electrode Fz were utilized for the final statistical analyses. In line with the consistent findings related to MMN stimuli examined in SZ patients, we chose to extract only pitch deviance MMN amplitudes for later statistical analyses because they are particularly sensitive to pathological brain excitation across different brain disorders and are specifically correlated with the magnitude of grey matter loss in frontal cortex regions in schizophrenia patients [14]. Tone-length and volume-deviant difference waveforms as a function of group can be viewed in Supplementary Figure S1.

## 3. Results

### 3.1. Patients versus Healthy Controls

**Figure 2:**
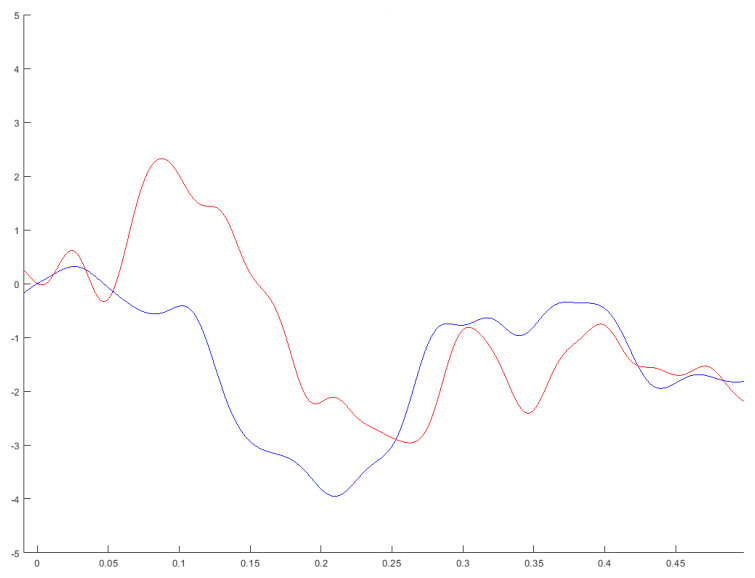
Grand averaged difference waveforms for changes in pitch under frontal Fz electrode in schizophrenia patients (red line) versus healthy controls (blue line). The *X* axis indicates a 0.5 s window post-stimulus with a 0.01 s pre-stimulus time window. *Y* axis represents the MMN amplitudes (5 to −5 µV). MMN difference waveforms were not statistically different.

After reviewing the score distributions of averaged MMN peak amplitudes/latencies obtained from the MMN difference waveforms and WM accuracy/RT scores within each group, we noted one outlier score (more than 2 SDs from the mean) within the HC MMN amplitude distribution and one outlier score within the SZ MMN amplitude distribution. In order to maximize group homogeneity, both outlier scores were discarded from further MMN amplitude statistical analyses. As expected, results indicated significant differences in WM accuracy scores between the groups, where SZ patients (M = 73.75. SD = 25.97) were significantly less accurate (*t*(17.4) = 2.64, *p* = 0.028) than HC (M = 96.5, SD = 14.17). There were no significant differences in MMN peak-amplitudes and latencies between patients and HC, although marginal differences in MMN peak-amplitudes approached significance (*t*(19.85) = −1.8, *p* = 0.087). As observed in Figure 2, grand average pitch-deviant MMN difference waveforms indicated a non-significant attenuated MMN in the SZ group versus HC. Although MMN peak latencies were slower in the SZ group, they did not significantly differ between SZ and HC (*p* = 0.55, two-tailed, Mann-Whitney U test). WM performance and MMN amplitudes/latencies were unrelated to chlorpromazine dose equivalents of patients’ ongoing antipsychotic medication (see Supplementary Table S1).

### 3.2. Relationship between MMN, Working Memory, and Clinical Status

**Figure 3:**
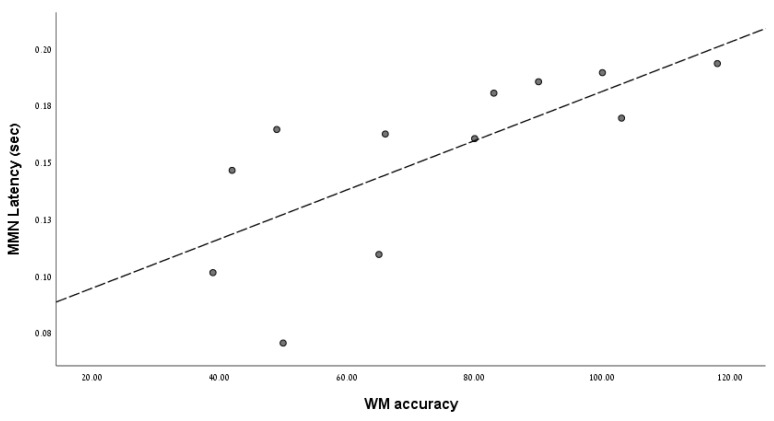
MMN Latencies are significantly related to WM accuracy only in schizophrenia patients. The X axis represents working memory (WM) accuracy, the maximum total accuracy score is 126 (number of hits). Y axis represents MMN peak amplitude latencies.

**Figure 4:**
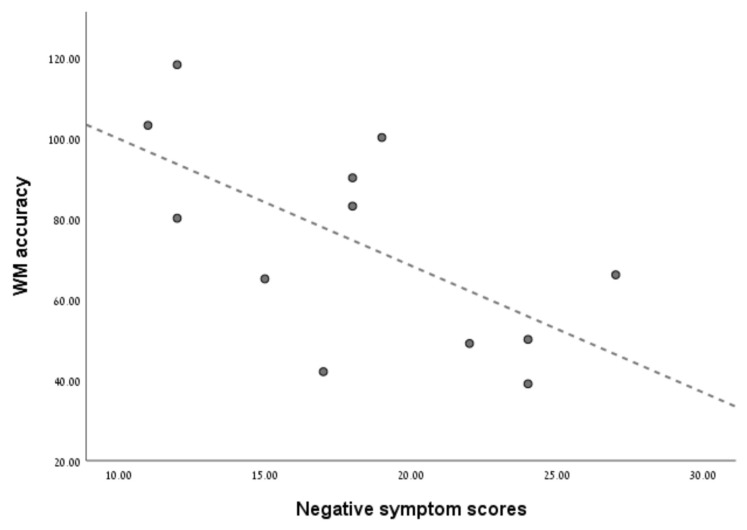
WM accuracy scores are related to negative symptom severity in the SZ group. The figure illustrates a significant relationship between WM accuracy and negative symptom severity in schizophrenia patients. The *Y* axis represents working memory (WM) accuracy; the maximum total accuracy score is 126 (number of hits). The *X* axis represents negative symptom severity scores ranging from a minimum score of 7 to a maximum score of 49.

MMN peak-amplitudes were unrelated to WM accuracy and RTs in both groups. However, a remarkably strong correlation was found between MMN peak-amplitude latencies and WM accuracy (Spearman’s rho = 0.82, *p* = 0.001), indicating that when MMN latencies are longer, WM accuracy is higher. This relationship was absent in HC (Spearman’s rho = 0.22, *p* = 0.48). See Figure 3 to review this unique significant relationship between WM accuracy and MMN latencies found in the SZ group. Next, step-wise multiple regression analyses were employed to evaluate whether MMN and WM variables could serve as possible significant predictors (MMN amplitude/latencies, WM accuracy/RTs) of clinical status scores (e.g., PANSS scores, positive and negative symptom scores, and MMSE scores) in the SZ group. Regression analysis (RA) incorporating these neurocognitive variables as predictors of total PANSS scores (i.e., illness severity) revealed that, unlike MMN variables and WM accuracy, only WM RTs remained a significant predictor of total PANSS scores (*R* = 0.61, *F*(1,9) = 5.33, *p* = 0.04). RA for predicting negative symptoms incorporating the same predictors indicated that only WM accuracy remained a significant predictor of negative symptom severity (*R* = 0.64, *F*(1,9) = 6.29, *p* = 0.033). See Figure 4 to review the relationship between WM accuracy and negative symptom scores. RA predictor variables were found to be unrelated to positive symptom scores and general psychopathology scores. Figure 4 illustrates the relationship between WM accuracy and negative symptom severity scores, indicating that as WM accuracy decreases, negatives symptom severity increases. MMSE scores were exclusively related to WM accuracy (*R* = 0.61, *F*(1,9) = 5.48, *p* = 0.044). None of the MMN variables were related to schizophrenia symptom severity or global cognitive function, as measured by PANSS and MMSE respectively.

## 4. Discussion

In the current study, EAP in SZ patients was associated with efficient WM performance; particularly, prolonged MMN latencies were related to better WM accuracy. This neurocognitive association was not observed in the HC group. In contrast to previous investigations indicating that impaired MMN and negative symptoms may result from reduced NMDA receptor activity [12,17], our results shed light on a novel relationship between EAP timing associated with NMDA plasticity and executive attentional control in schizophrenia [13]. This pattern of findings in SZ patients has previously been partially supported in other standard WM tasks [12,17] that are heavily dependent on either auditory or visual-spatial sensory processing. However, this is the first time that this type of association was revealed for executive verbal WM scores requiring multi-modal DLPFC engagement considered to reflect increased DLPFC control (i.e., frontal alpha functional inhibition) of temporarily stored domain-general information in interference-rich conditions [2,5,10,18,19]. Therefore, we suggest that in highly demanding verbal WM tasks that impose increased involvement of multidimensional memory storage and prefrontal functional inhibition over competing modality-specific WM associations, unlike HC, SZ patients may fail to engage their DLPFC networks to functionally inhibit irrelevant WM associations possibly affected by insufficient echoic WM activation (in turn possibly reflected by shorter MMN latencies) prior to initiating a response. 

In light of the current findings, it is suggested that DLPFC-mediated functional inhibition of irrelevant modality-specific networks may be disrupted in SZ due to “slower” or less efficient auditory cortex change-detection network reactivity. Thus, DLPFC-mediated functional inhibition of modality-specific cortical networks is possibly delayed (due to slower MMN latencies), as indicated by significantly impaired WM performance [10]. Hence, although NMDA receptor excitability is attenuated in SZ patients versus healthy controls, slower reactivity in NMDA receptor excitability within modality-specific networks in SZ may be detrimental to prefrontal functional inhibition, which could be quantified by examining anterior EEG alpha activity during online and offline WM periods [5,10], particularly during pre-retrieval periods that precede successful WM responses [5]. Accordingly, we extend and support previous findings indicating that certain hypoactive modality-free prefrontal networks in SZ lead to compromised top-down control of verbal WM representations [2,5]. In support of previous MMN findings in SZ patients [24], the current results indicated that prolonged early auditory cortex timing excitability within temporal-frontal pre-attentive neural networks was associated with better executive WM performance, particularly in schizophrenia patients. Moreover, our results confirmed an association between MMN latencies and executive attention functioning in SZ [5,9], which prior studies have failed to confirm [12,24].

Finally, in order to highlight the importance of specific DLPFC-related top-down processing and sensory abnormalities in SZ, our findings indicated that unlike MMN parameters, executive WM accuracy was exclusively related to higher levels of global cognitive functioning in schizophrenia. Furthermore, observed deficits in MMN generation in schizophrenia patients in the current study were not associated with schizophrenia symptom domains. In contrast, WM accuracy and RTs were significantly related to negative symptom severity and total illness severity, respectively. Our preliminary findings imply that unlike MMN parameters, noting DLPFC-mediated EA performance parameters in SZ patients can be instrumental in predicting overall clinical status. Additionally, it is important to note that previous studies showing an association between MMN peak amplitudes and executive functioning scores in SZ patients [14,17] (not replicated in the current study) possibly disregarded the fact that most of the standardized cognitive tasks used in the clinical-research setting rely on modality-specific DLPFC-response-selection mechanisms [17], which depend mainly on visual-spatial WM storage, rather than auditory processing or phonological loop activity associated with verbal WM storage [9].

### Study Strengths and Limitations

In light of the exploratory nature of the current investigation, and since the current findings are based on a relatively small sample size of SZ patients and a limited number of cognitive measures, it will be critical to replicate our findings in order to demonstrate a consistent relationship between EA performance and MMN latencies and similar correlational directionality in larger SZ samples and versus other psychiatric populations. It would also be prudent to examine the predictability of MMN parameters versus executive attention parameters over time. Addressing these limitations in future studies will enhance the confirmation of our current preliminary findings. Moreover, it is imperative to cross-validate observed correlations in other cognitively challenging EA tasks that demand rapid multimodal WM storage [2,5] and evaluate the presence of this association in other psychiatric populations suffering from dysfunctional prefrontal cortex activity and MMN abnormalities. The main contribution of the current investigation is that it sheds light on a unique neurocognitive association in SZ patients, specifically in cognitively challenging conditions. Finally, the current study supports the superiority of EA parameters versus MMN parameters in predicting overall clinical status in SZ.

## 5. Conclusions

In conclusion, we suggest that novel treatment approaches in SZ should target DLPFC EA-networks that participate in synchronizing different sources of information. Interventions that modulate MMN latencies may improve the reactivity of modality-specific association cortex networks, and by doing so may facilitate coherent verbal WM representation in SZ patients prior to goal-directed responses [8]. In support, recent findings revealed that left DLPFC transcranial direct stimulation (tDCS) add-on treatment in chronic SZ patients significantly alleviated symptom severity versus sham stimulation and acutely improved verbal WM performance versus baseline WM performance [5]. Importantly, in light of the current findings, directing clinicians’ attention towards the development of specific tDCS treatment interventions in SZ [25] that attain long-term-improvements in executive WM functioning will likely lead to a significant alleviation of illness severity, and more importantly, to a reduction in negative symptom severity, which is considered a predictor functional outcome in SZ [12].

## Data Availability

The data presented in this study are available on request from the corresponding author. The data are not publicly available due to clinical and ethical restrictions related to clinical-research data confidentiality.

## Supplementary Materials

The following supporting information can be downloaded at: https://www.mdpi.com/article/10.3390/brainsci12020212/s1 [26], Table S1: Clinical characteristics of chronic schizophrenia patients. Figure S1: MMN difference waveforms for tone length and tone volume deviance.
