# Supplementary material for: Early Auditory Processing Predicts Efficient Working Memory Functioning in Schizophrenia
Source: Brain Sci. 2022 Feb 3;12(2):212. doi: 10.3390/brainsci12020212 (PMC8870168; doi:10.3390/brainsci12020212)
Supplement: Supplementary file 1 [file brainsci-12-00212-s001.zip › brainsci-1555523-supplementary.pdf]

Patients	Diagnosis	Duration (months)	Medication	Chlorpromazine dose equivalents mg/day
1	SZ	60	Zuclopenthixol (Valproate)	15
2	SA	120	Zuclopenthixol, Clotiapine (Lithium)	10
3	SZ	420	Risperidone	5
4	SZ	324	Haloperidol	3
5	SA	408	Halidol	6.5
6	SZ	444	Olanzapine	3.5
7	SZ	600	Perphenazine, Sulpiride	5.5
8	SZ	216	Aripiprazole	30
9	SZ	96	Zuclopenthixol	50
10	SA	228	Aripiprazole, Haloperidol, Clotiapine (Valproate)	40
11	SZ	169	Amisulpride, Risperidone	8
12	SZ	120	Risperidone	4

Table S1: Clinical characteristics of chronic schizophrenia patients. SZ = schizophrenia, SA = schizoaffective disorder, Duration = Disease duration from onset to study participation, Medication = current antipsychotic medication regimen of each schizophrenia patient. In parenthesis are names of adjunctive agents to antipsychotic medications such as anticonvulsants and mood stabilizers. Chlorpromazine dose equivalents for comparing different antipsychotic medications were calculated according to Thippaiah et al (2021) [26].

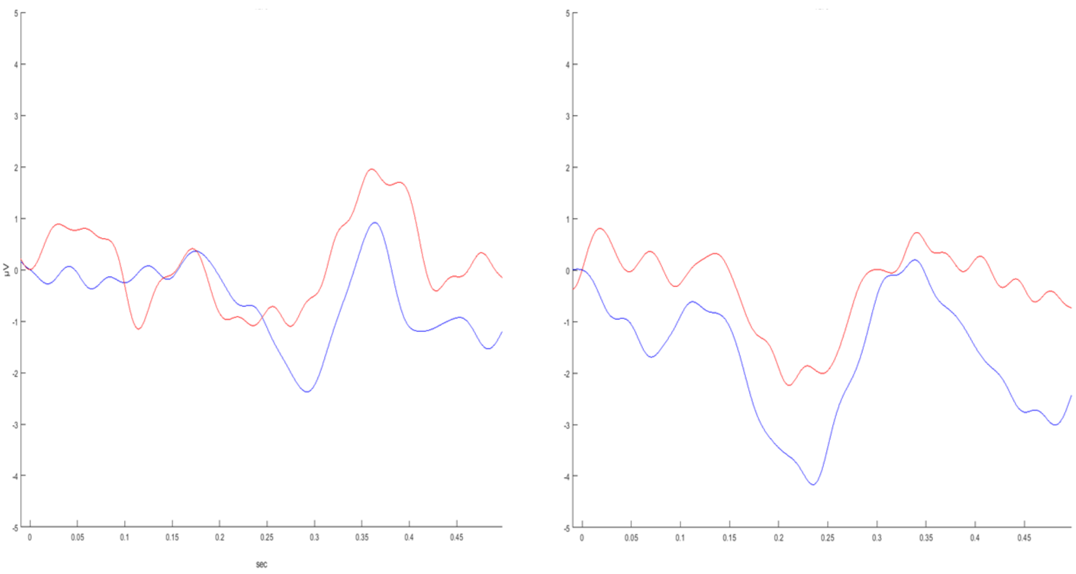

Figure S1. Tone length and volume deviant difference-waveforms as a function of group. Tone length deviance difference-waveforms (left side) and tone volume deviance (right side) in the schizophrenia group (red line) versus healthy controls (blue line).
